# The impacts of first line highly active antiretroviral therapy on serum selenium, cd4 count and body mass index: a cross sectional and short prospective study

**DOI:** 10.11604/pamj.2013.15.97.2524

**Published:** 2013-07-12

**Authors:** Adeolu Oladayo Akinboro, Olaniyi Onayemi, Olugbenga Edward Ayodele, Ayodele David Mejiuni, Adeniran Samuel Atiba

**Affiliations:** 1 Department of Internal Medicine, Ladoke Akintola University of Technology and LAUTECH Teaching Hospital, Ogbomoso, Oyo State, Nigeria; 2 Department of Dermatology and Venereology, Obafemi Awolowo University and OAUTHC, Ile-Ife, Osun State, Nigeria; 3 Bullsbrook Medical Practice, Perth, Australia; 4 Department of Chemical Pathology, Ekiti state University and Ekiti State Teaching Hospital, Ekiti State, Nigeria

**Keywords:** HIV/AIDS, Selenium, CD4 count, BMI, HAART

## Abstract

**Introduction:**

The relationship that exists between body weights, serum selenium and immunological markers of HIV/AIDS continue to provoke more researches in the recent times. The objectives of this study were to examine baseline body mass index, CD4 count and serum selenium and to prospectively assess the impacts of HAART on same parameters 48 weeks post HAART among HIV patients.

**Methods:**

A cohort comprising 140 newly diagnosed HIV positive were prospectively studied. Anthropometric measurements, serum selenium and CD4 count were assessed at diagnosis and 48 weeks post HAART.

**Results:**

The mean age for patients was 35±8.8 years; 68% was female. Patients’ mean weight was 56.79±10.22kg, BMI; 21.59±3.53, serum selenium; 0.55 ± 0.45µmol/L and CD4 count; 288.36 ± 232.23 at the baseline. At diagnosis, 47 (33.6%) were in stage 1, 49 (35.0%) in stage 2, 26 (18.6%) and 18 (12.9%) were in stage 3 and 4 respectively. Similarly, most patients had normal body mass index, 94 (67.14%), 26 (18.57%) were underweight, (12.86%) were overweight and two (1.43%) were obese at diagnosis. At 48 weeks post HAART, the mean weight, BMI, serum selenium and CD4 count were significantly increased.

**Conclusion:**

HAART repleted CD4 count and serum selenium, Post HAART overweight was associated with lesser CD4 count reconstitution and selenium repletion. A renew call for weight monitoring in HAART era.

## Introduction

The highly active anti retroviral therapy (HAART) has changed the outlook of Human Immunodeficiency Virus /Acquired Immune Deficiency Syndrome patients (HIV/AIDS) worldwide and in sub Sahara Africa (SSA) [1, 2]. Since the first discovery in 1996, HAART is known to confer certain benefits which include: reduction in morbidity, improvement in the quality of patients’ life, sustained suppression of viral load and enhance immune cell recovery [2]. However, over times, there are matters arising from nutritional stand point: current reports from certain quarters had signified an on-going epidemic of obesity, clustered of metabolic risk factors and its associated reduction in immune cell recovery in the era of HAART [3–5]. Similarly, the deficiency of certain micronutrients with anti-oxidative stress properties such as selenium has also been notable in this region. Responsible factors include: ecological factors, chronic household food deficiency, economic sabotage and the HIV itself [1, 2, 6]. The malnutrition, weight loss and CD4 count depletion which was the norm pre HAART still exists in the developing countries in the HAART era [7]. The potential benefits of ameliorating these nutritional deficiencies have become a subject of many scientific studies in recent times [1, 3]. The attendant complications arising from these nutritional abnormality outcomes in the HAART era have become subject of intense researches. Such studies are scarce in Nigeria. There is an increasing need to know how HIV patients on HAART in SSA fares on HAART and how this nutritional anomaly takes its toll on them in HAART era.

Studies have suggested the linkage between weight loss induction, depletion of CD4 count and of serum selenium in HIV/AIDS. The suggested pathway include: oxidative stress induction [8, 9], and activation of NF-κb leading to cellular apoptosis of T lymphocytes and increased HIV replication [10]. The outcome of chronic oxidative stress induction in HIV include: the release of tumour necrosis factor - alpha, (TNF α) and the interleukins (ILs) which are responsible for both the weight loss and selenium depletion [10, 11]. It is also known that HIV incorporates host selenium into the viral seleno - proteins thereby depleting its host of selenium [10].

Reports from Nigeria regarding this problem are scanty [12–16], both in pre HAART and the post HAART era. Existing studies are cross sectional, and prospective studies investigating impacts of HAART on weight CD4 count and serum selenium are grossly inadequate. Since HAART induces viral suppression, we thus hypothesized that the common pathway that produces weight loss, CD4 count and selenium depletion would have been suppressed. This study therefore examined the relationship between body weight (body mass index -BMI), immune status (CD4 count), and selenium concentration at diagnosis and evaluated the impacts of HAART on same parameters among HAART-naive HIV/AIDS after 48 weeks.

## Methods

This was a cross-sectional and prospective study consisting of 140 newly diagnosed HIV-positive patients and 140 apparently healthy HIV sero-negative individuals. Both populations were randomly recruited into the study. Every 3^rd^ newly diagnosed HIV positive individuals that sought attention in the clinic were so recruited and examined at the clinic dedicated to PLWHA in Ladoke Akintola University of Technology Teaching Hospital (LTH), Osogbo, Nigeria.

The purposes and procedures of the study were explained to the participants. Newly diagnosed HIV-positive patients and HIV sero negative controls who gave informed written consent were included. The control population consisted of age and sex-matched apparently healthy men and women recruited from the same local Government area. Other inclusion criteria for the subjects and controls included: age between 18 - 64 years, absence of history of smoking [17], non-use of selenium-containing supplements prior to or during the study period and absence of any acute illness that needed urgent medical or surgical intervention. The age 64 years was chosen as the upper limit because, selenium status is known to decrease slightly among healthy elderly people compared to younger adults [18]. The study protocol was approved by the Ethical Board of studies of the LTH, Osogbo.

**Sample collection and laboratory evaluation:** Human immunodeficiency virus was diagnosed by using two different enzyme immunoassays: Determine HIV-1/2 (Abbott Diagnostic Division, Belgium/Luxemburg) and Uni-Gold HIV Kit (Trinity Biotech, Wicklow Bay, Ireland). Patients reactive to two antibody screening tests were considered positive and recruited into the study. We collected 10 mls of fasting venous blood from antecubital fossa of both the patients and control population: 5mls of the fasting whole blood was collected into a sterilized plain tube for selenium estimation and 5mls was stored in EDTA bottle for CD4+cell assessment. CD4+cell count was estimated using FAC Scan flow Cytometry (CyFlow SL Green, Partec GmbH Münster, Germany 2006) from whole blood samples kept in EDTA bottles.

**Estimation of serum selenium:** Selenium concentration was estimated at Science Research Laboratory of Rufus Giwa Polytechnic, Owo, Ondo state of Nigeria. Five millilitres of whole blood sample from the study participants was left on laboratory table to clot, retract and was centrifuged (Centaur MSE centrifuge machine made in Fisions, England) at 3,500 rpm for 5 min after which the serum was separated and stored at - 20° until assayed. The concentration of selenium was assayed after wet ashing by hydride generating atomic absorption spectrometry [19, 20], using Atomic Absorption spectrophotometer - Model 210 VGP, / Buck Scientific, East Norwalk, CT, USA 2004) as previously described [19, 20]. Standard laboratory selenium solutions containing known selenium content were used as reference solution to control for the quality of the analysis. The laboratory analyst was blinded as to which samples were subject or control. Two measurements were made for each sample and average of the observations was recorded as research data.

**Clinical assessment:** The socio demographic characteristics of the study population were obtained using a structured questionnaire. Anthropometric assessment of the subjects and controls was made on the day of HAART commencement. The weight of all subjects and controls were obtained while patients were wearing light clotting, using weighing scale weight was recorded to the nearest 0.1kg; height was obtained using standiometer with patients looking straight forward, head in horizontal plane, feet apposed, with shoulder relaxed and arm placed at the side as described by Lee and Niemann in 2003 [21]. Height was measured and recorded to the nearest 0.1cm. Body mass index (BMI) was calculated from the formula: weight (kg) / height^2^ (M). Patients were categorized into weight groups as: underweight (BMI < 18.5 kg/m^2^), normal weight (BMI 18.5-24.9kg/m^2^), overweight (BMI 25-29.9 kg/m^2^), and obese BMI ≥30.0 kg/m^2^ according to WHO criteria [22]. Patients were screened for tuberculosis with careful physical examination, chest examination, chest x-ray and sputum tests for AAFB on three occasions. Patients with tuberculosis were commenced on anti-tuberculosis drugs and excluded from follow up cohort. All the patients that qualified for highly active anti-retroviral therapy (HAART), i.e WHO stages 4A, B, C, 1C, 2C, 3C and 3B, if symptomatic of the disease, after baseline assessment were commenced on first line therapy, according to the WHO Guidelines on the Treatment of HIV/AIDS for adult and adolescent which was adopted as the National guideline [23]. The first line therapies used included: Lamivudine + Zidovudine + Nevirapine combination, and Lamivudine + Zidovudine + Efavirenz combinations [22].

**Procedures at follow up:** The follow-up of the cohort was done monthly and at 6 months. The prescribed 1^st^ lines HAART were renewed during each monthly visit. During each visit, patients were also attended to by trained nurses, adherence counsellors, and pharmacist to ensure adherence to medication. None of the study participants took selenium supplement during the follow-up period. Counselling was given monthly on the need to adhere to HAART medications, and pill counting was done regularly to ensure at least 95% adherence. Instruction was also given to all participants on eating balanced diet and drinking potable water. At 6 months after the baseline, the patients’ weights were reassessed, and samples were taken for CD4+ cell count and serum selenium, to assess the impact of HAART on selenium levels and the CD4 count. Those who failed to return for their scheduled appointment were visited at home or contacted on cell phone.

**Statistical analysis:** Continuous and categorical variables are displayed as means ± SD and percentages respectively. Differences between continuous variables were analyzed using Student's t test. Chi square test was used to assess differences between categorical variables. Differences among ≥ 2 groups were compared using the Analysis of Variance (ANOVA) test. The mean of the difference between baseline CD4+cell counts, serum selenium, weight, BMI and their values six months post HAART were analyzed using paired student t-test. All p values were two tailed and p values < 0.05 were considered to be statistically significant. All data analyses were done using the Statistical Programme for Social Sciences (SPSS) version 16.0 (SPSS Chicago Inc., IL, and U.S.A.)

## Results

One hundred and fourty HIV positive patients were recruited at diagnosis. The mean age of the patients’ population was 35.00 ± 8.80years. Eighty percent (119) of the study population were below the age of 45years while 21 (15.0%) were older than fourty five years. Ninety six (68.6%) of the recruited HIV patients’ population were females. While 74 (52.9%) of the patients’ were out of marriage (widowed or divorce) at the time of diagnosis, 66 (47.1%) were married. Among the HIV/AIDS patients, 11 (7.9%) were illiterate, 54 had at least primary education and 75 (53.6%) had at least secondary education. In the present cohort of HIV patients, 68 (48.6%) were predominantly petty trader, others (51.4%) were non trader.

The HIV patients were classified according to WHO Clinical staging, 47 (33.6%) were in stage one, 49 (35.0%) stage two, 26 (18.6%) stage three, and 18 (12.9%) in WHO stage four. The mean height, weight, BMI, CD4 Count and serum selenium are as shown in Table 1. At the baseline, the patients were stratified according to WHO BMI classification. Most patients had normal weight, 94 (67.14%), 26 (18.57%) were underweight, (12.86%) were overweight and two (1.43%) were obese at diagnosis. The prevalence of Tuberculosis in the cohort was 20.0% and 112 (80.0%) had no clinical Tuberculosis. At baseline, 34 (61.8%) was commenced on Nevirapine based first line HAART, and 21 (38.2%) had Efaverenz based HAART.

**Table 1:** The baseline demographics and clinical characteristics of the study population

Demographics and clinical parameters	N (%)
**Mean Age ± SD (years)**	35.00 ± 8.8
Below 45years	119 (85.0)
Above 45 years	21(15.0)
Gender (female)	96(68.6)
**Marital Status**	
Presently out of Marriage	74 (52.9)
Presently Married	66 (47.1)
**Education**	
Illiterate	11 (7.9)
At least Primary education	54 (38.6)
At least Secondary Education	75 (53.6)
**Occupation**	
Predominantly Petty Traders	68 (48.6)
Non Traders	72 (51.4)
**WHO Clinical staging**	
Clinical Stage 1	47(33.6)
Clinical Stage 2	49(35.0)
Clinical Stage 3	26(18.6)
Clinical Stage 4	18(12.9)
**Anthropometric and Clinical Characteristics**	
Baseline median Height (range)	1.62 ±0.08
Baseline median Weight (range)	56.79±10.22
Baseline median BMI (range)	21.59±3.53
Baseline median CD4 count (range)	288.36±232.23
Baseline Serum Selenium (range)	0.55±0.45
**WHO BMI Classification**	
Underweight	26(18.6)
Normal weight	94(67.1)
Overweight	18(12.9)
Obese	2(1.4)
**Pulmonary Tuberculosis (PTB)**	
With PTB	28(20.0)
Without PTB	112(80.0)
**HAART**	
AZT_+_ 3TC _+_ NVP	34(61.8)
AZT_+_ 3TC _+_ EFV	21(38.2)

Weight < 18.5kg – Underweight, 18.5 -24.9kg- Normal weights, 25 –29.9 kg - Over weights, > 30.0 kg- Obese, N= number, % - Percentage. WHO – World Health Organisation. SD – Standard Deviation. BMI – Body Mass Index. HAART- Highly Active antiretroviral therapy. AZT-Zidovudine, 3TC-Lamivudine, NVP-Nevirapine, EFV- Efavirenz. CD4 count -cell/mm^3^, Serum Selenium -µ±mol/L

**Figure 1:**
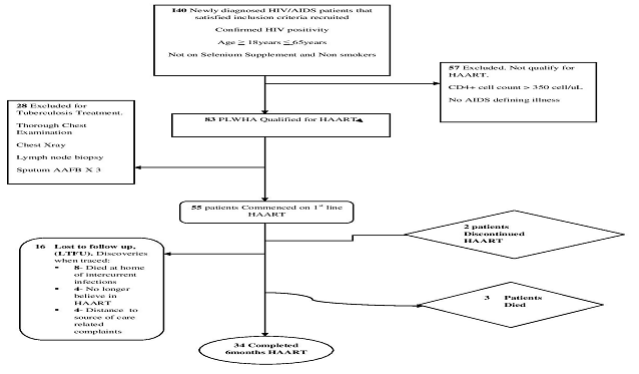
Follow up and outcome distribution of HIV positive patients.

The schematic representations of the follow up events were as shown in Figure 1. One hundred and forty newly diagnosed HIV sero positive patients that fulfilled the inclusion criteria were recruited. Of that sum, fifty seven patients (40.7%) were excluded from HAART, because they had CD4 count ≥350 cells/ mm3 and or were without any AIDS-defining illness and eighty three patients (59.3%) qualified for initiation of first line HAART. Of the 83/140 (59.3%) who qualified for HAART, 28/140 (20.0%) had clinically proven tuberculosis and were excluded from the follow-up cohort to start anti-TB drugs. Therefore, 55/83 (66.3%) of the patients that qualified for HAART were started on first line HAART and were subsequently followed up for six months. However, only 34/ 55(61.82%) completed the monthly follow up schedules. Two patients (3.63%) discontinued medication despite counselling and 16/55(29.09%) were lost to follow up (LTFU) and did not return to care during the study despite visitation and phone calls. Of the 16/55 cases that were LTFU, (8/16) 50.0% fell sick, refused to come to hospital and died at home, 4/16 (25.0%) could not keep to the schedule appointment because they were living in a far distance, and the other 4/16 (25.0%) did not return to care because they did not believe in HAART as a cure of their disease any more, Figure 1. We also observed no differences in the mean of age, sex ratio, mean weight, BMI, serum selenium and CD4 count of the LTFU group and the overall HIV/AIDS population.

After six months of follow up while on HAART, the outcomes of the 34 patients that completed HAART was assessed. Thirty one (91.2%) had positive response to HAART with increased CD4+ cell count, while 3/34 (8.8%) had decreased in CD4 count from the baseline evaluation. Also, 29/34 (85.3%) gained weight, 2/34 (5.9%) had reduction in weight, and weight was static in 3/34 (8.8%). In the same vein, 29/34 (85.3%) had increased level of serum selenium and 5/34(14.7%) had decreased selenium compared to baseline values. Three patients died during the course of follow-up, giving a mortality rate of 5.45%.

We observed a significant rise (p< 0.001 (Table 2). Similarly, at the baseline (Pre HAART), the means ± SD of serum selenium rose from 0.45 ± 0.33µmol/L to 1.03± 0.59 with a mean increase of - 0.57 µmol/L inn serum selenium. The observed increase was statistically significant, p< 0.001 (Table 3).

**Table 2:** Mean weight, bmi, serum selenium and cd4 count ± SD at baseline and 48 weeks post HAART initiation for 34 HIV/AIDS patients that completed followed up

	Baseline	48 weeks Post HAART	Pair mean difference	p-value
Weight(kg)	54.05±8.45	60.21±8.99	-6.15	<0.001
BMI	20.65± 2.89	23.00±3.03	-2.34	<0.001
Serum Selenium (µmol/L)	0.45 ± 0.33	1.03± 0.59	-0.57	<0.001
CD4 Count (cells/mm^3^)	121.50 ± 84.83	265.79 ± 151.84	-144	<0.001

**Table 3:** The pre HAART and post HAART distribution of mean patients’ weight, body mass index, serum selenium and CD4 count ± SD according to who classification of body mass index

Variables	Underweight	Normal	Overweight	Obese	p- value
**Pre HAART**					
Mean weight, n = 140	45.93±6.10	56.60±6.59	69.61±8.03	91.00±9.90	<0.001
Mean BMI, n = 140	17.29±1.20	21.45±1.80	27.13±1.63	33.97±1.06	<0.001
Mean CD4 count, n = 140	111.77±92.44	328.26±242.27	303.06±200.27	577.00±106.06	<0.001
Mean Serum selenium n = 140	0.37±0.35	0.59±0.47	0.61±0.46	0.57±0.45	0.170
**Post HAART**					
Mean Weight, = 34	53.54±7.57	63.27±8.12	66.25±3.18	-	0.006
Mean BMI, n = 34	20.64±1.61	23.71±2.57	28.87±1.66	-	<0.001
Mean CD4 count, n = 34	263.38±153.20	221.64±148.36	134.33±148.98	-	0.290
Mean Serum selenium, n= 34	0.77±0.49	1.18±0.60	1.08±0.55	-	0.320

BMI - Body Mass Index Weight < 18.5kg – underweight, 18.5 -24.9kg- normal weights, 25 –29.9 kg - Over weights, > 30.0 kg- Obese, n= number

Patients were further stratified according to WHO classification of body mass index. The study demonstrated the underweight category had lowest mean BMI ± SD, lowest CD4 count, and lowest serum selenium concentration. The CD4 count increased across the weight stratifications with slight reduction in the overweight group than the normal weight patient. However, the highest CD4 count was observed among the obese group, p for the distribution was < 0.001. Mean while, serum selenium concentration progressively increased from underweight to overweight category, with reduction in serum selenium concentration in the obese category, the observed distribution of selenium concentration was not statistically significant, p =0.170.

At 48 weeks post HAART, there was overall increased in the mean weight, BMI across categories, serum selenium concentration, and CD4 count beyond the pre HAART levels. However, observation showed the distribution of CD4 count reduced progressively from underweight category to the overweight category, p = 0.207. Serum selenium concentration however increased from underweight category to the overweight category insignificantly, p = 0.378. No patient was classified into the obese category post HAART in this study. There was a significant positive correlation between baseline serum selenium and CD4 count (r = 0.2, p < 0.001), baseline serum the selenium and BMI, (r = 0.3, p < 0.001).

## Discussion

This study demonstrated that the use of first line HAART comprises of 2 NRTI such as Zidovudine and Lamivudine as backbone, with either of NNRTI like Efavirenz or Nevirapine was associated with significant mean increase in the body mass index, serum selenium and CD4 count among HAART- naive HIV/AIDS patients after 48weeks of at least 95% adherent HAART.

At the baseline, there was a progressive increase in the CD4 count as body weight increases, in difference to Crum-Cianflone et al et al [3, 4], that showed no such association at the time of diagnosis. But in similarity with their study, the recovery of CD4 count after HAART was least with overweight category and was highest with the underweight category of patients after 48weeks of HAART commencement. This finding further supported the suggested potential detrimental effect of excessive weight gain on immune reconstitution in the HAART era [3, 4]. It must be noted that this finding was found in a population of patients with overt baseline malnutrition and with least tendencies to excessive weight gain. Therefore, having higher BMI was only temporarily beneficial for CD4 constitution at baseline or time of diagnosis among HIV positive patients. Post HAART, this initial conferred advantage was found detrimental as increased weight was associated with a progressive reduction in CD4 count reconstitution, as found by Crum-Cianflone et al. studies [3, 4]. Suggested hypothetic mechanisms responsible for reduced CD4 count among overweight HIV patients in HAART era include: low grade systemic inflammation being induced by elevated C- reactive protein [3], or inadequate concentration of HAART in body tissue due to mal-distribution of HAART [3, 4]. Our findings also reinforces the need for continuous advocacy for close monitoring of body weight and its maintenance in the HAART era and support the suggestion of further studies that will assess the role oxidative stress and inflammatory markers HIV/AIDS patients. [3, 4].

The pair means increase in weight was 6.15kg, which is close to median 5.0kg (range, 1.5 - 10.7kg) in review of studies conducted in 14 African countries by Akileswaran et al. [24] Also, the pair means increase of CD4 count was 144cells/mm^3^ also falls within previously documented range of 74 - 440 cells mm^3^ [24]. While 91.2% of our patient showed positive response to HAART with increased CD4 count, More & Keruly [25] in 2007 demonstrated comparable 92% CD4 count increase. We demonstrated an inverse association between weight loss and CD4 count, weight loss was prominent clinically in advance HIV disease as found in previous studies [7, 13] [24–27].

Post HAART, most of the follow-up cohort subject (85.3%), gained variable amount of weight, although we did not establish whether this increase was due to increase in the lean body mass or fat. The reported epidemic of overweight and obesity post HAART may not be the usual experience in the SSA, and this is not unexpected because of the prevailing malnutrition been caused by chronic household insufficiency, poor socio-economic condition and overall ailing economy. However, the small number of patients in the follow up cohort may not be sufficient for this generalization. The prevalence of metabolic syndrome has also been found to be comparatively lower (12.7-21.0% using different criteria) among HIV/AIDS patients in this region than outside the region. [28]. In the same vein serum there was a progressive increase in serum selenium concentration as weight categories increase from underweight to overweight as previously documented [24, 29, 30], but the present study shows obesity was associated with reduce selenium concentration when compared with the other weight groups at the point of diagnosis. Similar trend was observed post HAART, as selenium repletion was lowest among patients in the overweight category when compared with other weight groups. In further comparison with earlier studies, lower levels of selenium concentration have been documented among immune competent adult with obesity and or metabolic syndrome compared to non obese [31]. Majid et al. [31] demonstrated overall higher selenium concentration among immunocompetent obese patients but observed depletion in selenium level with increasing features of metabolic syndrome.

The link between weight loss, chronic induction of acute phase reactants and low serum anti oxidants levels and HIV has been long suggested. Elevated catabolic chemokines such as TNF-α, IL-1, IL-6 and interferon which are thought to be responsible for inflammation induction have been documented in AIDS patients without evidence of any secondary infection [7, 9, 30, 32]. The resulted hyper catabolic state induced by these chemokines has been linked to C-reactive protein, inflammation, changes in hormone production, increase neutrophils number and noticeable alteration in serum levels of trace elements such as selenium and other antioxidants [7, 11]. The prevailing low weight, BMI and serum selenium profile at the baseline can be traced or attributed to this fact. Over duration of 48 weeks only 61.82% of the follow up cohort were retain in care, was also in line with previously documented range of 53% to 100%. The causes of LTFU include death at home due to opportunistic infection, inability to keep up with clinic attendance and loss faith in HAART.

We have some limitations in this study. First, viral load assessment was not done; hence it was difficult to characterized the patients who failed to respond to HAART. Also difficulty associated with getting this category of patients enrolled into this type of study will continuously restrict this study type to the source of care in our environment. The large number of people lost to follow up, markedly reduced the follow up cohort.

## Conclusion

Low category of BMI was associated with lower CD4 count and serum selenium concentration among HIV/AIDS patients at diagnosis. The use of first line HAART was associated with CD4 count reconstitution and serum selenium repletion post HAART. Post HAART overweight was associated with lesser CD4 count reconstitution and serum selenium repletion. Our finding reinforced the advocacy for weight monitoring and its maintenance in the HAART era while research continues to unravel role of selenium in HIV/AIDS, its use as adjuvant micronutrient may alleviate micronutrient malnutrition.
